# UNDERSTANDING AGING IN TERMS OF PHYSIOLOGICAL STATES

**DOI:** 10.1093/geroni/igz038.2297

**Published:** 2019-11-08

**Authors:** Alexander Mendenhall, Nikolay Burnaevskiy, Soo Yun, Bryan Sands

**Affiliations:** University of Washington, Seattle, Washington, United States

## Abstract

The “network” of homeostatic systems fails in distinct ways in individual isogenic animals during the aging process. We believe that understanding these distinct physiological states, the transitions between them, and how they relate to homeostatic system functions will allow us to better affect change in the aging process. Work in yeast showed that fixing an initial system failure, loss of vacuole acidification capacity, could increase cellular lifespan. Here we showed how the long-lived physiological state conferred by high expression of the hsp-16.2 promoter based lifespan/penetrance biomarker correlates with differences in the expression of other genes, and the structure and function of lysosomes. We found that vacuole acidification failure is not a major initial proximal cause of aging in C. elegans – at least not in their intestine cells.

